# A Case Report of Unilateral Syphilitic Uveitis: A Diagnostic Challenge and the Role of Point-of-care Ultrasound

**DOI:** 10.5811/cpcem.1435

**Published:** 2024-01-09

**Authors:** Susana Gutierrez-Luke, Thijs Wolf, Kyle Green, Philip Graber

**Affiliations:** * University of Rochester Medical Center, Department of Emergency Medicine, Rochester, New York; † University of Rochester Medical Center, Department of Ophthalmology, Rochester, New York

**Keywords:** *case report*, *syphilis*, *syphilitic uveitis*, *ocular ultrasound*

## Abstract

**Introduction:**

Syphilis presents with diverse clinical manifestations, posing challenges for diagnosis, especially in the fast-paced emergency department (ED) setting.

**Case Report:**

We report a rare case of unilateral syphilitic uveitis in an individual who had been sexually abstinent for 13 years. Using ocular point-of-care ultrasound in the ED, we successfully diagnosed this uncommon ocular manifestation.

**Conclusion:**

Our case highlights the diagnostic challenges of ocular syphilis and emphasizes the crucial nature of timely identification. Collaborative efforts with specialties such as ophthalmology are essential in overcoming these challenges.

Population Health Research Capsule What do we already know about this clinical entity?

*Syphilis can present with ocular manifestations including uveitis, which is difficult to diagnose especially in the ED.*

What makes this presentation of disease reportable?

*Unilateral vitritis is a unique presentation of syphilis that can be seen on ocular point-of-care ultrasound (POCUS), which can be used as an adjunct when making this diagnosis.*

What is the major learning point?

*Ocular syphilis is difficult to diagnose. Ocular POCUS can aid with clinical suspicion. A multidisciplinary approach in collaboration with ophthalmologists is important.*

How might this improve emergency medicine practice?

*Emergency physicians can use POCUS to aid with diagnosis and clinical findings for ocular syphilis.*

## INTRODUCTION

The incidence of syphilis has increased over the past two decades to 12.7 cases per 100,000, up from a low of 2.1 cases per 100,000 in 2001, representing a six-fold increase.^1^ Syphilis, caused by the spirochete *Treponema pallidum*, is known as “the great mimicker” due to its various clinical presentations. Although rare, ocular syphilis can lead to devastating consequences. Timely administration of effective treatment is crucial in preventing irreversible visual impairment. Prompt evaluation of acute ocular pain and vision loss is particularly essential for monocular patients. Ocular point-of-care ultrasound (POCUS) is a rapid and dynamic imaging modality that emergency physicians can use to aid in diagnosis. We present the case of a 47-year-old man who, despite sexual abstinence for 13 years, was diagnosed with ocular syphilis with the assistance of POCUS.

## CASE REPORT

A 47-year-old male was brought to the emergency department from his primary care physician’s office after complaining of blurred vision and pain in his left eye for the prior four weeks. The patient initially dismissed these symptoms as a headache or optic neuritis, which he had previously experienced due to his medical history of multiple sclerosis. However, his vision gradually deteriorated over time, with the appearance of floaters and a significant increase in pain two weeks prior to presentation. On physical examination, the patient’s left eye had a visual acuity of 20/60 and a superior temporal field cut. Unfortunately, his right eye had previously suffered vision loss due to optic neuritis and could only perceive light. The intraocular pressure in the left eye was 14 millimeters of mercury (mm Hg), while the right eye’s pressure was 11 mm Hg. The patient’s neurologic examination was otherwise unremarkable.

**Image 1.**
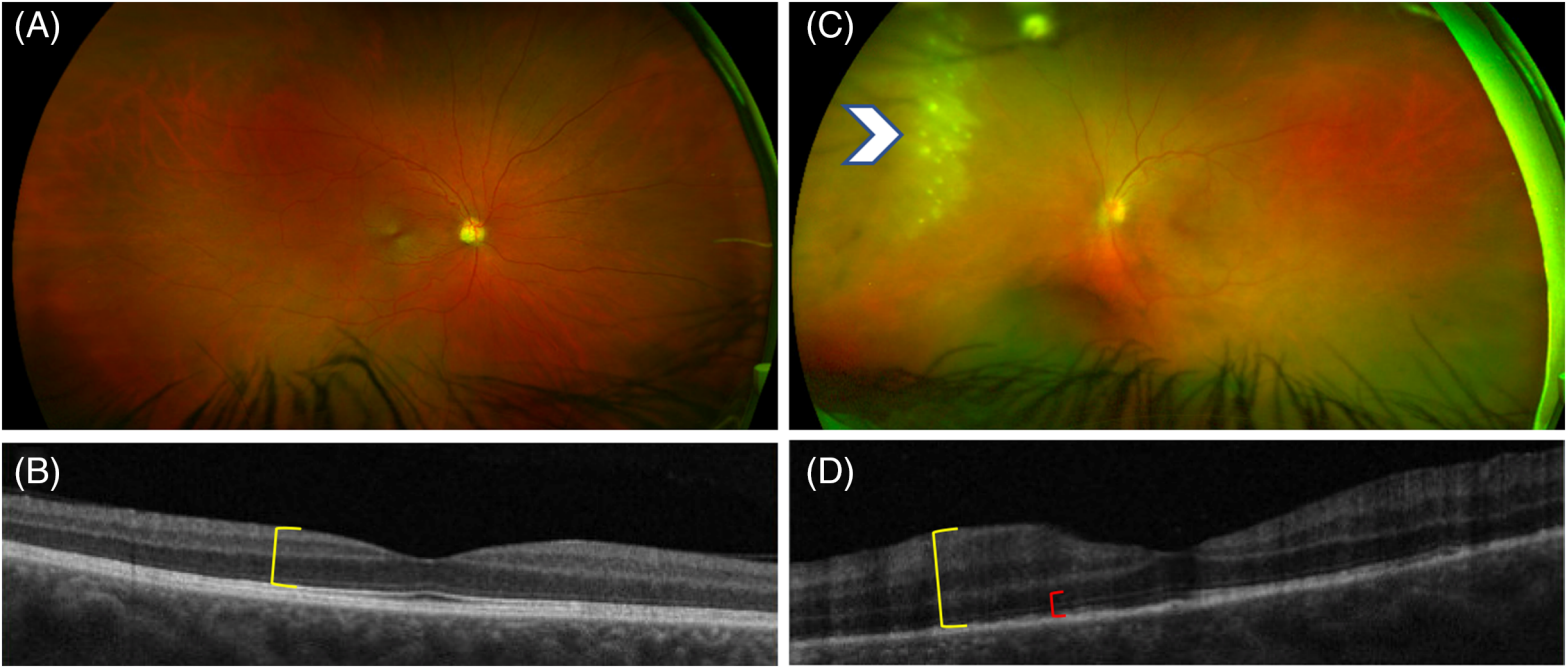
Ophthalmic imaging of syphilitic retinitis. (A) Normal appearing fundus of the right eye captured with wide-field photography. (B) Accompanying optical coherence tomography of the macula in the right eye with slight thinning (yellow bracket) but otherwise normal-appearing retinal architecture. (C) Fundus photo of the left eye with area of superonasal retinal whitening and scattered focal white opacities (arrowhead) representing syphilitic retinitis (neurosyphilis). (D) Relative macular thickening of the left eye (yellow bracket) with an irregular photoreceptor layer (red bracket, (compare to right eye).

**Image 2.**
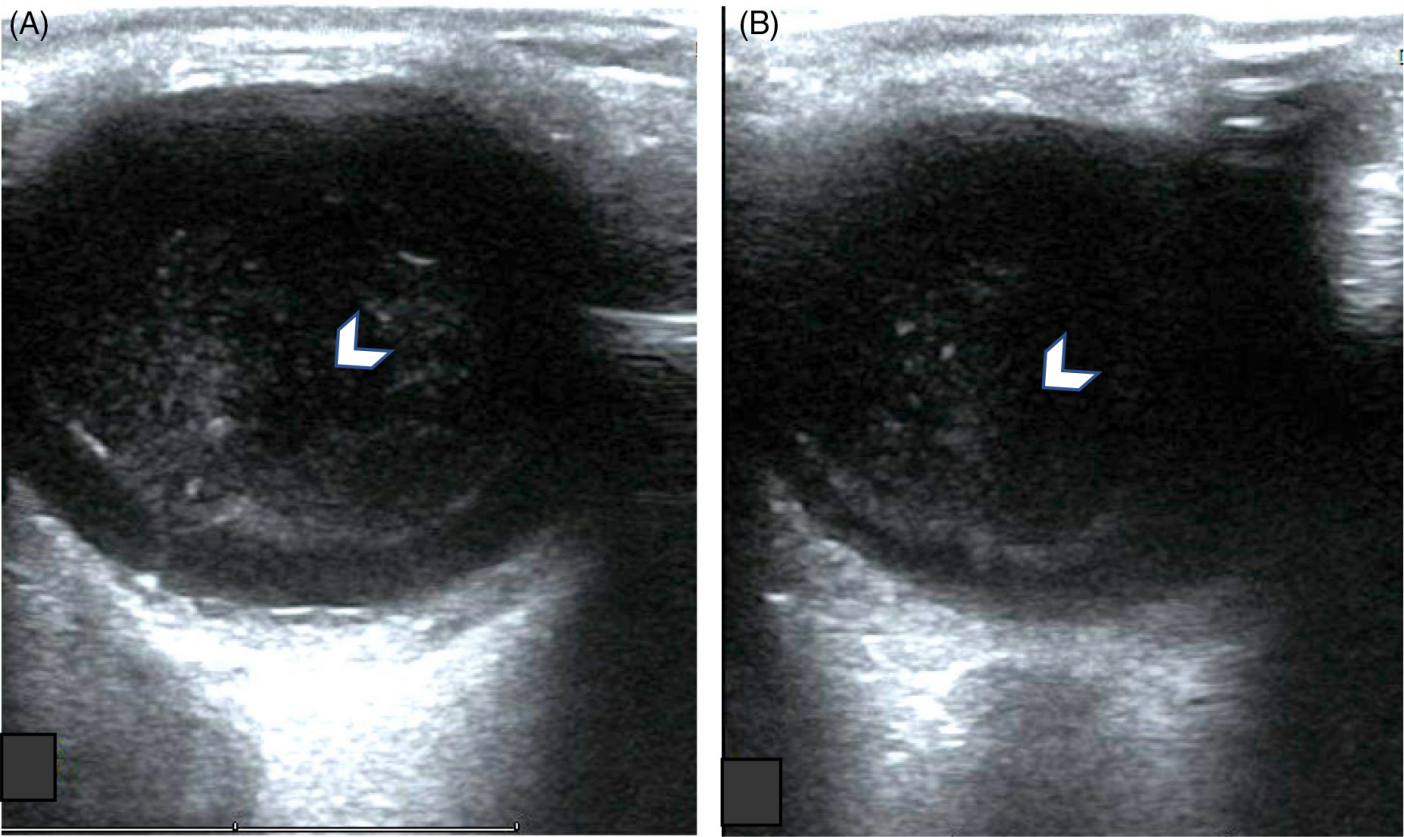
Ocular point-of-care ultrasound using the linear probe. (A) Transverse view of the orbit. (B) Sagittal view of the orbit. Subtle echogenic material is seen floating in the vitreous (arrowheads).

The ophthalmologic exam revealed left conjunctival injection and ciliary flush. Dilated ophthalmic examination showed peripheral retinal whitening in the superonasal area of the left eye, as well as high-grade inflammation in the anterior chamber (3+ cell) and vitreous cavity (2+ cell) (Image 1). Laboratory testing revealed a rapid plasma reagin titer of 1:256, with the patient testing negative for HIV. He was treated with intravenous penicillin 4 million units every 4 hours for 14 days. Ultrasonography of the eye revealed echogenic particles within the vitreous, with a possible undulating membrane that moved freely and swirled on dynamic exam (Image 2). The patient’s retina was attached, and the diameter of the optic nerve was within normal limits.

## Using Ocular Point-of-Care Ultrasound

To perform ocular POCUS the patient is placed supine; a tegaderm film is placed over the eye to eliminate air bubbles and then copious aqueous gel is spread over the closed eyelid. A linear transducer is used in both sagittal and axial planes of the eye with dynamic scanning. To get accurate views the clinician should hold the transducer like a pencil and brace their fifth digit over the patient’s face gently to be mindful of how much pressure is being applied to the eye. Reducing the gain will show the walls of the globe and optic nerve sheath perfectly. Increasing the gain enables the contents of the vitreous body to be studied.

## DISCUSSION

Syphilis has earned its reputation as the “great mimicker” due to its ability to cause symptoms that may imitate various diseases.^2^ Ocular syphilis presents a challenge because of its diverse range of clinical features—it can be unilateral or bilateral, acute or chronic, and affect all or only certain anatomical locations.^3^ Point-of-care ultrasound can help distinguish between conditions that may be difficult to differentiate otherwise, owing to its portability, expedient bedside interpretation, and non-invasive nature. Syphilis is classified into four stages: primary, secondary, latent, and tertiary syphilis.^4^ While ocular symptoms can occur at any stage of the disease, research indicates that they are most frequently seen in secondary syphilis.^5^

Ocular manifestations account for 2–10% of infectious uveitis cases worldwide^6^ and can also be linked to neurosyphilis, with patients often presenting with eye pain, vision loss, and neurological changes.^7^ Retinitis, as a form of retinal involvement, qualifies as neurosyphilis since the retina is an extension of the central nervous system. If left untreated, the disease can progress to blindness, making it crucial to remain vigilant in certain high-risk populations. Men who have sex with men have been reported to have the highest transmission rates.^1^ Diagnosing syphilitic eye infection can be challenging, as its presentation can overlap with many other conditions. A diagnosis typically requires an ophthalmic examination, combined with serological tests specifically for syphilis. Therefore, a thorough history, physical examination, and a high degree of clinical suspicion are critical for clinicians.

Strong indications for serological testing include a history of sexually transmitted diseases, high-risk sexual behavior, or inflammatory findings on ophthalmologic examination.^8^ The patient presented here had evidence of unilateral vitritis on POCUS, decreased visual acuity, vision loss, and ocular pain; he was immediately referred to a tertiary center to see an ophthalmologist. After the diagnosis, he was promptly treated with antibiotics, leading to resolution of his symptoms. Although nonspecific, sonographic imaging can aid in the differential diagnosis and follow-up of inflammatory and non-inflammatory pathologies of the posterior segment. Many posterior uveitis syndromes are due to underlying infections such as toxoplasmosis, syphilis, toxocariasis, tuberculosis, cytomegalovirus retinitis, and ocular histoplasmosis syndrome.^9^ In addition, sonographic imaging can help detect complications of ocular syphilis, such as retinal detachments, optic neuritis, vitreous hemorrhage, or vitreous detachment. A multidisciplinary approach, particularly in collaboration with ophthalmologists, is crucial.

## CONCLUSION

Ocular syphilis presents a diagnostic challenge due to its diverse clinical manifestations, often leading to misdiagnosis. Early diagnosis and treatment are crucial to prevent vision loss and other neurologic complications. A comprehensive evaluation that includes a thorough medical history, physical examination, ophthalmic examination, laboratory testing, and sonographic imaging can aid in the accurate diagnosis of ocular syphilis. Collaboration among primary care physicians, emergency physicians, and ophthalmologists is essential for the successful management of this condition.

## References

[1] Centers for Disease Control and Prevention. Sexually Transmitted Disease Surveillance. Available at: https://www.cdc.gov/std/statistics/2020/2020-SR-4-10-2023.pdf. Accessed June 29, 2023.

[2] Deschenes J Seamone C Baines M. The ocular manifestations of sexually transmitted diseases. Can J Ophthalmol. 1990;25(4):177–85. 2191758

[3] Bollemeijer JG Wieringa WG Missotten TO et al. Clinical manifestations and outcome of syphilitic uveitis. Invest Ophthalmol Vis Sci. 2016;57(2):404–11. 26848879 10.1167/iovs.15-17906

[4] Janier M Hegyi V Dupin N et al. 2014 European guideline on the management of syphilis. J Eur Acad Dermatol Venereol. Dec 2014;28(12):1581–93. 25348878 10.1111/jdv.12734

[5] Eslami M Noureddin G Pakzad-Vaezi K et al. Resurgence of ocular syphilis in British Columbia between 2013–2016: a retrospective chart review. Can J Ophthalmol. 2020;55(2):179–84. 31889521 10.1016/j.jcjo.2019.11.002

[6] Bertrand PJ Jamilloux Y Ecochard R et al. Uveitis: autoimmunity… and beyond. Autoimmun Rev. 2019;18(9):102351. 31323361 10.1016/j.autrev.2019.102351

[7] Kiss S Damico FM Young LH. Ocular manifestations and treatment of syphilis. Semin Ophthalmol. 2005;20(3):161–7. 16282150 10.1080/08820530500232092

[8] Koundanya VV Tripathy K. Syphilis ocular manifestations. *StatPearls*. 2022. Available at: https://www.ncbi.nlm.nih.gov/pubmed/32644383. Accessed June 29, 2023. 32644383

[9] Morais FB Arantes TEFE Muccioli C et al. Ultrasonographic characteristics of active ocular toxoplasmosis. Arq Bras Oftalmol. 2019;82(4):317–21. 31038555 10.5935/0004-2749.20190063

